# Prevention of High-Fat Diet-Induced Hypercholesterolemia by *Lactobacillus reuteri* Fn041 Through Promoting Cholesterol and Bile Salt Excretion and Intestinal Mucosal Barrier Functions

**DOI:** 10.3389/fnut.2022.851541

**Published:** 2022-03-11

**Authors:** Mengyao Lu, Jin Sun, Yuning Zhao, Haowen Zhang, Xinyue Li, Jingbo Zhou, Hongyang Dang, Jidong Zhang, Wenjing Huang, Ce Qi, Duo Li

**Affiliations:** ^1^Institute of Nutrition and Health, Qingdao University, Qingdao, China; ^2^Department of Cardiology, The Affiliated Hospital of Medical College, Qingdao University, Qingdao, China; ^3^Department of Paediatrics, The Affiliated Hospital of Medical College, Qingdao University, Qingdao, China

**Keywords:** *Lactobacillus reuteri*, high-fat diet, gut microbiota, cholesterol, bile acid

## Abstract

**Objectives:**

*Lactobacillus reuteri* Fn041 (Fn041) is a probiotic isolated from immunoglobulin A coated microbiota in the human breast milk of Gannan in China with a low incidence of hypercholesterolemia. This study aims to explore the role and mechanism of Fn041 in preventing hypercholesterolemia caused by a high-fat diet in mice.

**Methods:**

C57BL/6N mice were fed a low-fat diet or a high-fat diet and gavage with Fn041 and *Lactobacillus rhamnosus* GG (LGG) for 8 weeks.

**Results:**

Both Fn041 and LGG prevented the occurrence of hypercholesterolemia, liver and testicular fat accumulation. In addition, a high-fat diet causes intestinal dysbiosis and mucosal barrier damage, which is associated with hypercholesterolemia. Fn041 prevented the high-fat diet-induced reduction in alpha diversity of intestinal microbiota and intestinal mucosal barrier damage. Fn041 treatment significantly increased fecal total cholesterol and total bile acids.

**Conclusions:**

Fn041 prevented hypercholesterolemia by enhancing cholesterol excretion and mucosal barrier function.

## Introduction

Chronic exposure to a high-fat diet can lead to an accumulation of cholesterol in the blood (1). An elevated plasma total cholesterol (TC) level is a recognized risk factor for coronary heart disease, atherosclerosis, and strokes (2). Successful management of cholesterol metabolism disorders can effectively prevent these diseases (3). There is growing evidence that gut microbiota dysbiosis strongly influences the development of cholesterol metabolism (4, 5). Statins are currently the most effective cholesterol-lowering drugs. However, their long-term use can have side effects such as hepatotoxicity and muscle toxicity (6). Therefore, there is a need to develop nutritional interventions with no side effects.

In the past decade, several researchers have confirmed that a 70 kg man contains about 3.8 × 10^13^ bacteria and interact with each other (7). Probiotics are defined by the World Health Organization as live microorganisms that provide health benefits to the host when given in sufficient amounts and are presently being evaluated for their efficacy in lowering TC and low-density lipoprotein cholesterol (LDL-C) levels in humans (8). Some probiotics, mainly *Lactobacillus* and *Bifidobacterium*, have potential cholesterol-lowering benefits in the gastrointestinal tracts of mammals (6, 9, 10). Early animal studies have shown that short-term prophylaxis and intervention with low doses of specific *Lactobacillus reuteri* can exert cholesterol-lowering effects (11). A recent meta-analysis compiled 15 foreign randomized controlled trials on lipid modulation using probiotics and showed that consuming *L. reuteri* significantly reduced total serum cholesterol and LDL-C levels (12). A population-based trial indicated that yogurt containing *L. reuteri* CRL 1098 reduces LDL-C and TC levels in patients with high cholesterol levels (13). Healthy hypercholesterolemic adults can reduce their cholesterol levels by taking capsules containing *L. reuteri* NCIMB 30242 (14). We previously isolated *L. reuteri* Fn041 from the breast milk samples of healthy mothers in the Gannan agricultural and pastoral areas of Gansu Province (15). The prevalence of hypercholesterolemia in the population of this region is lower than that in the highly industrialized areas of the East and South (16). The acid tolerance, bile salt tolerance, hydrophobicity, and mucus adhesion of Fn041 were higher than those of the typical probiotic *Lactobacillus rhamnosus* GG (LGG) (unpublished data). The former strain showed a high bile acid resistance, suggesting its potential role in the regulation of lipid metabolism. In our previous study, we found that Fn041 alleviates the effect of high-fat diet-induced dyslipidemia in mice (17); therefore, we further increased the fat supply ratio to observe whether Fn041 could prevent dyslipidemia and compared its results with those of the typical probiotic LGG to determine whether the cholesterol-lowering efficacies and mechanisms of the two strains were the same.

Based on previous studies, the following hypotheses have been proposed regarding the anti-cholesterol effects of probiotics: *Lactobacillus* degrades bile salts in the intestine; it also promotes the following: excretion of bile salt degradation products from the feces (18, 19), the production of short fatty acids (20), cholesterol assimilation (21), the coprecipitation of cholesterol with deconjugated bile and cholesterol conversion to coprostanol (22), cholesterol transport by intestinal epithelial cells for excretion (23), and cholesterol synthesis in the liver (24–26). Among these hypotheses, we are most concerned about the conversion of uncoupled bile acids into secondary bile acids by colonic microbes to control serum cholesterol levels (19).

This study aimed to explore whether Fn041 and LGG can prevent dyslipidemia and mucosal barrier damage caused by a high-fat diet. We also investigated the effects of strains Fn041 and LGG on intestinal microbiota and how they lower cholesterol levels *in vivo*.

## Materials and Methods

### Diets and Animals

Sixty male C57BL/6N mice weighing 18–21 g (6 weeks old) were provided by Vital River Laboratory Animals Co., Ltd. (Beijing, China). All animal experimental procedures were performed in accordance with the Guidelines for Care and Use of Laboratory Animals of Qingdao University and approved by the Animal Ethics Committee of the Affiliated Hospital of Qingdao University (Approval No. QYFYWZLL25869). The mice were fed in the Experimental Animal Center of Qingdao University with a 12 h light/dark cycle, constant temperature (22 ± 1°C), and constant humidity (50 ± 5%). All mice were given free access to water and a standard diet. After a week of acclimation, the mice were randomly divided into the following five groups (*n* = 12/group): LF, low-fat diet-fed mice; LF+Fn041, low-fat diet-fed mice treated with Fn041; HF (60% energy from fat), high-fat diet-fed mice; HF+Fn041, high-fat diet-fed mice treated with Fn041; HF+LGG, high-fat diet-fed mice treat with LGG. Fn041 and LGG were suspended in saline, and 1 × 10^9^ colony-forming units (CFU) were administered daily via gavage. The same volume (100 μL) of saline was administered daily to low-fat and high-fat control mice. The probiotic treatment lasted for 8 weeks. The mice were weighed weekly; weight gain rate = (final week weight - starting weight)/starting weight. The compositions of the low-fat and high-fat diets are provided in the Supplementary Table S1.

### Analyses of Biochemical Indicators

At the end of the animal experiment, the mice were anesthetized after 16 h of fasting and sacrificed via decortication. After the mouse was anesthetized, blood samples were collected using eyeball removal method. Blood samples were collected and isolated via centrifugation (Sigma3K15, Germany) at 1500 × *g* and 4°C for 10 min. TC, triglycerides (TG), LDL-C, and high-density lipoprotein cholesterol (HDL-C) levels in plasma, and catalase and malondialdehyde concentrations in liver tissue homogenates were measured using assay kits (Nanjing Jiancheng Bioengineering Institute, Nanjing, Jiangsu, China). Plasma levels of tumor necrosis factor α (TNF-α), interleukin 6 (IL-6), lipopolysaccharide (LPS), and lipopolysaccharide-binding protein (LBP) were quantified using enzyme-linked immunosorbent assay kits (Xiamen Huijia Biotechnology Co., Ltd, China). The enzyme-linked immunosorbent assay kits were also used to detect the contents of TC and total bile acid in feces (Xiamen Huijia Biotechnology Co., Ltd, China).

### Histological Analysis of Fat and Liver Tissues

Fresh liver and testicular fat tissues were fixed with 4% paraformaldehyde and embedded in paraffin. Tissue sections of 5 μm thickness were prepared for hematoxylin and eosin and Oil Red O staining. Images were captured using a light microscope. The calculations for relevant indexes are as follows: Testicular fat index = testicular fat weight/body weight, perirenal fat index = perirenal fat weight/body weight, and liver index = liver fat weight/body weight.

### Determination of Intestinal Permeability

The mice were subjected to fasting for 6 h and gavaged with 150 μL of 80 mg/mL 4.4 kDa fluorescein isothiocyanate-dextran (FD4; Sigma, St Louis, MO). Plasma was collected at 4 h post-gavage, diluted 1:5 (v/v) in phosphate-buffered saline, and transferred to a black opaque-bottom 96-well plate. Fluorescence was measured spectrophotometrically in 96-well plates (excitation, 485 nm; emission, 530 nm).

### 16S rRNA Gene Sequencing Analysis

Total genomic DNA was extracted from the ileum content using a QIAamp DNA Stool Mini Kit (Qiagen, Hilden, Germany). The DNA concentration was monitored using an Equalbit dsDNA HS Assay Kit. Then, two highly variable regions of prokaryotic 16S rDNA, including V3 and V4, were amplified using 20–30 ng DNA as a template using polymerase chain reaction (PCR) primers. The V3–V4 regions of the 16S rRNA gene were amplified via PCR using universal primers (forward: 5′- ACTCCTACGGGAGGCAGCA-3′ and reverse: 5′-GGACTACHVGGGTWTCTAAT-3′). Thermal cycling consisted of 94°C for 3 min, followed by 30 cycles at 94°C for 45 s, 56°C for 1 min, and 72°C for 1 min, with a final extension at 72°C for 10 min. Then, a linker with an index was added to the end of the PCR product of 16S rDNA via PCR for next-generation sequencing. Library concentrations were measured using zymography and quantified at 10 nM. PE250/FE300 double-end sequencing was performed using Illumina MiSeq (Illumina, San Diego, CA, USA) instruments, and sequence information was read using MiSeq Control Software. The sequences were joined and depleted of barcodes, and sequences < 200 bp and those with ambiguous base calls were removed. The final obtained sequences were used for operational taxonomic unit clustering, and sequence clustering was performed using VSEARCH (1.9.6; sequence similarity was set to 97%); Silva 132 was the 16S rRNA reference database used for the comparison. The raw data of 16S rRNA gene libraries generated during this study are publicly available at the Sequence Read Archive portal of NCBI under accession number PRJNA747157.

### Real-Time Quantitative PCR

Total RNA was extracted from the liver and intestinal tissues and then reverse transcribed into cDNA. Real-time PCR was used to detect mRNA expressions using β-actin as an internal reference. Genes studied for liver included cholesterol-7α-hydroxylase (*Cyp7a1*) with a forward primer (5′ to 3′) of AGCAATGAAAGCAGCCTCTGA and a reverse sequence (5′ to 3′) of TGATGCTATCTAGTACTGGCAGGT, as well as liver X receptor alpha (*Lxr*) with a forward primer sequence (5′ to 3′) of TGGAGACGTCACGGAGGTACA and a reverse sequence (5′ to 3′) of CAGCTCATTCATGGCTCTGGA. Ileum *Slc10a2* was detected with a forward primer sequence (5′ to 3′) of TAGATGGCGACATGGACCTCA and a reverse sequence (5′ to 3′) of CCCGAGTCAACCCACATCTTG. The amplification conditions were as follows: 95°C for 30 s, 1 cycle; 95°C for 5 s, 60°C for 32 s, 40 cycles; 95°C, 15 s, 60°C, 60 s, and 95°C, 1 s, 1 cycle.

### Feces and Ileum Content Collection

Feces were collected one day before the mice were sacrificed, and the mice were placed on a disposable tablecloth. After the animals excreted, the fecal samples were immediately transferred to Eppendorf tubes and placed in liquid nitrogen tanks for quick freezing. On the day of sacrificing, the ileum was isolated, and the ileum contents were extruded with forceps.

### Statistical Analysis

The mean differences between the groups were analyzed using one-way analysis of variance followed by *post-hoc* Tukey's test or Kruskal–Wallis test. Data were statistically analyzed using SPSS software (version 23.0; SPSS Inc., Chicago, IL, USA) and expressed as mean ± standard error of the mean. The statistical significance was set at *p* < 0.05 or *p* < 0.01. For the gut microbiota taxon analysis, the Kruskal–Wallis rank test was performed, and *p*-values were adjusted for multiple comparisons using the false discovery rate. Based on the operational taxonomic unit analysis results, using random sampling, the sample sequences were flat. Shannon, Chao1 alpha diversity index via the R package, vegan (v2.5-6), and the differences in β-diversity were visualized via principal coordinates analysis plots and tested for inferences using a permutational multivariate analysis of variance (Adonis from the package vegan, with 999 permutations); ggplot was used to construct a heatmap. GraphPad Prism 8.0 was used for additional graphs.

## Results

### *L. reuteri* Fn041 Alleviates High-Fat Diet-Induced Hyperlipidemia and Fat Accumulation

**Figure 1:**
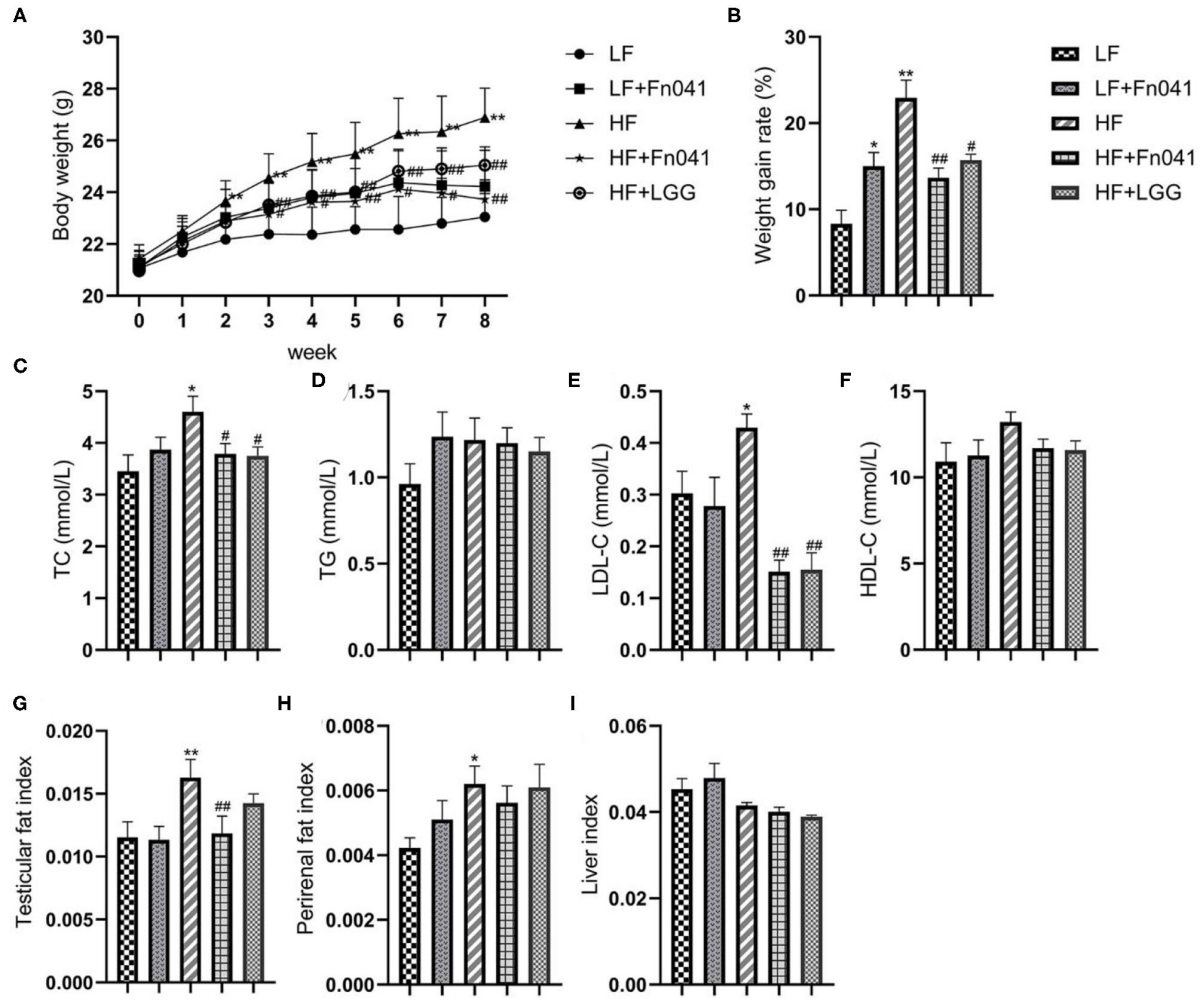
Effect of *Lactobacillus reuteri* Fn041 on body weight, fat accumulation, and serum biochemical indexes in the high-fat diet mice. **(A)** Weight change; **(B)** The weight gain rate; **(C)** TC; **(D)** TG; **(E)** LDL-C; **(F)** HDL-C; **(G)** Testicular fat index; **(H)** Perirenal fat index; **(I)** Liver index. Data are presented as mean ± SEM (*n* = 12/group); *p < 0.05, **p < 0.01 as compared with the LF group; ^#^*p* < 0.05, ^##^*p* < 0.01 as compared with the HF group. LF, low-fat diet group; LF + Fn041, low-fat diet group treated with *L. reuteri* Fn041; HF, high-fat diet group; HF + Fn041, high-fat diet group treated with *L. reuteri* Fn041; HF + LGG, high-fat diet group treated with *Lactobacillus rhamnosus* GG. HDL-C, high-density lipoprotein cholesterol; LDL-C, low-density lipoprotein cholesterol; TC, total cholesterol; TG, triglyceride.

After 8 weeks of high-fat diet feeding, the body weight of mice increased significantly, and the body weight of mice was 17.6% higher in the HF group than in the LF group. At the end of the experiment, the HF+LGG and HF+Fn041 groups had significantly lower weight gains throughout the experiment compared to the HF group (*p* < 0.05 or *p* < 0.01; Figures 1A,B, respectively). TC and LDL-C levels were significantly higher in the HF group than in the LF group. Compared with the HF group, both the Fn041 and LGG treatments significantly reduced TC (*p* < 0.05, Figure 1C) and LDL-C (*p* < 0.01, Figure 1E) levels. Plasma TG and HDL-C levels in both the LF and HF groups were identical to those of their counterpart groups (Figures 1D,F). Testicular fat (*p* < 0.01, Figure 1G) and perirenal fat (*p* < 0.05, Figure 1H) were significantly higher in the HF group than in the LF group. Testicular fat significantly decreased following the Fn041 treatment (*p* < 0.05, Figure 1G). Liver weights in both the LF and HF groups remained the same as those of their counterparts (Figure 1I).

### Effect of *L. reuteri* Fn041 on the Morphology of the Liver and Testicular Fat

**Figure 2:**
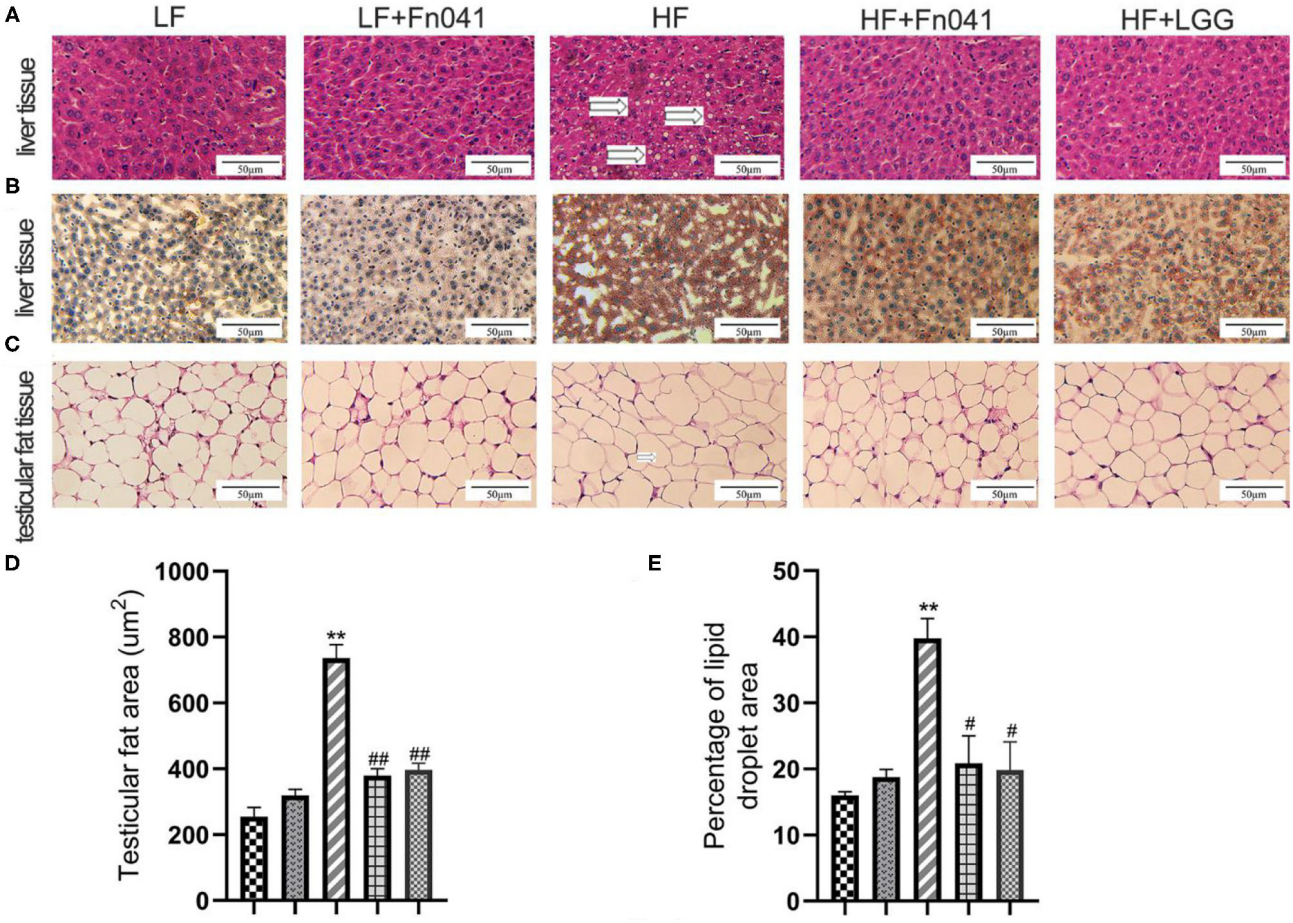
Effect of *Lactobacillus reuteri* Fn041 on the morphology of the liver and testicular fat. **(A)** H&E staining of the liver; **(B)** Oil Red O staining of the liver; **(C)** H&E staining of the testicular fat; **(D)** Adipocyte size (*n* = 5/group); **(E)** Percentage of lipid droplet area (*n* = 5/group). **p < 0.01 as compared with the LF group; ^#^*p* < 0.05, ^##^*p* < 0.01 as compared with the HF group. LF, low-fat diet group; LF + Fn041, low-fat diet group treated with *L. reuteri* Fn041; HF, high-fat diet group; HF + Fn041, high-fat diet group treated with *L. reuteri* Fn041; HF + LGG, high-fat diet group treated with *Lactobacillus rhamnosus* GG.

Inflammatory cell infiltration and lipid vacuolation were evident in the hematoxylin and eosin-stained liver sections of high-fat diet-fed mice (Figure 2A). The lipid accumulation in the liver of the HF group was higher than that of the other groups in the Oil Red O strained section (Figure 2B), and the lipid droplet area percentage in the liver of the HF group was significantly higher than that of the LF group. By comparison, lipid accumulation and lipid droplet area percentage in the livers of HF+Fn041 and HF+LGG groups were significantly lower than those in the HF group (*p* < 0.05 or *p* < 0.01, Figure 2E). According to the hematoxylin and eosin-stained adipose tissue section, the high-fat diet led to a pronounced expansion of adipocyte size compared with the LF group (Figure 2C). The adipocyte size of mice in the HF group was significantly larger than that in the LF group but was significantly reduced following the Fn041 and LGG treatments (*p* < 0.01, Figure 2D).

### Effects of *L. reuteri* Fn041 on Oxidative Stress in the Liver

**Figure 3:**
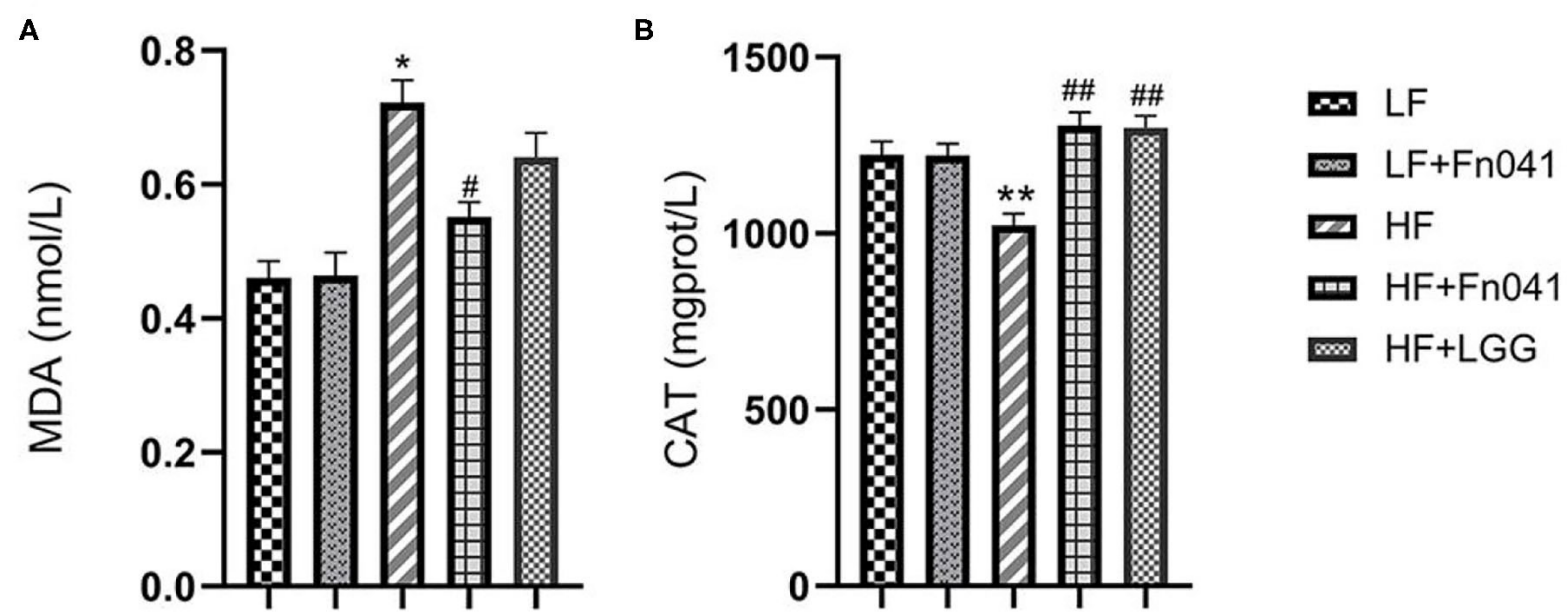
Effects of *Lactobacillus reuteri* Fn041 on oxidative stress in the liver. **(A)** MDA level; **(B)** CAT activity; Data are presented as mean ± SEM (*n* = 12/group); *p < 0.05, **p < 0.01 as compared with the LF group; ^#^*p* < 0.05, ^##^*p* < 0.01 as compared with the HF group. LF, low-fat diet group; LF + Fn041, low-fat diet group treated with *L. reuteri* Fn041; HF, high-fat diet group; HF + Fn041, high-fat diet group treated with *L. reuteri* Fn041; HF + LGG, high-fat diet group treated with *Lactobacillus rhamnosus* GG. CAT, Catalase; MDA, Malondialdehyde.

Liver malondialdehyde levels significantly decreased in the HF+Fn041 group compared to the HF group (*p* < 0.05, Figure 3A). Catalase activity levels in the liver of Fn041-treated and LGG-treated mice were higher than that in the HF group (*p* < 0.01, Figure 3B).

### Effects of *L. reuteri* Fn041 on Intestinal Permeability and Serum Endotoxin Levels

**Figure 4:**
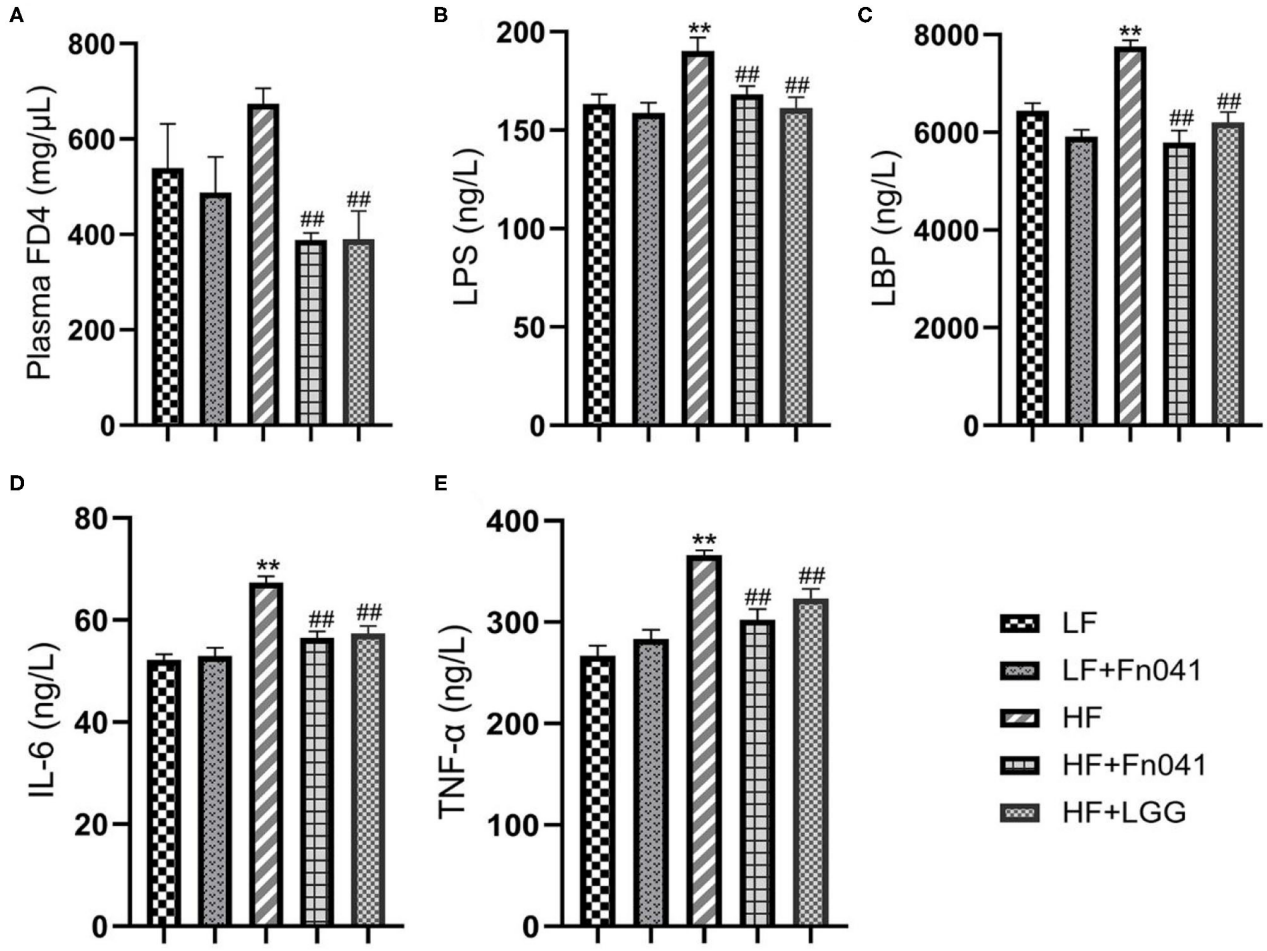
Effects of *Lactobacillus reuteri* Fn041 on intestinal barrier dysfunction and chronic inflammation. **(A)** Plasma FITC-dextran 4000 (FD4) concentrations (λex = 485; λem = 530 nm) measured 4 h after gavage (*n* = 5/group); **(B)** plasma lipopolysaccharide (LPS); **(C)** plasma lipopolysaccharide-binding protein (LBP); **(D)** plasma IL-6; **(E)** plasma TNF-α. Data are presented as mean ± SEM (*n* = 12/group); **p < 0.01 as compared with the LF group; ^##^p < 0.01 as compared with the HF group. LF, low-fat diet group; LF + Fn041, low-fat diet group treated wit*h L. reuteri* Fn041; HF, high-fat diet group; HF + Fn041, high-fat diet group treated with *L. reuteri* Fn041; HF + LGG, high-fat diet group treated with *Lactobacillus rhamnosus* GG.

Fluorescence FD4 measurements showed that FD4 values were significantly lower in the HF+Fn041 and HF+LGG groups than in the HF group (*p* < 0.01, Figure 4A). Plasma LPS and LPS-binding protein levels in the HF group were significantly higher than those in the LF group, whereas these levels were significantly lower in the HF+Fn041 and HF+LGG groups than in the HF group (*p* < 0.01, Figures 4B,C). We also examined the plasma levels of pro-inflammatory factors, including TNF-α and IL-6. In the HF group, plasma IL-6 and TNF-α levels were significantly higher than those in the LF group, and plasma IL-6 and TNF-α levels in the HF+Fn041 and HF+LGG groups were significantly lower than those in the HF group (*p* < 0.01, Figures 4D,E).

### Effect of *L. reuteri* Fn041 on Ileum Microbiota

**Figure 5:**
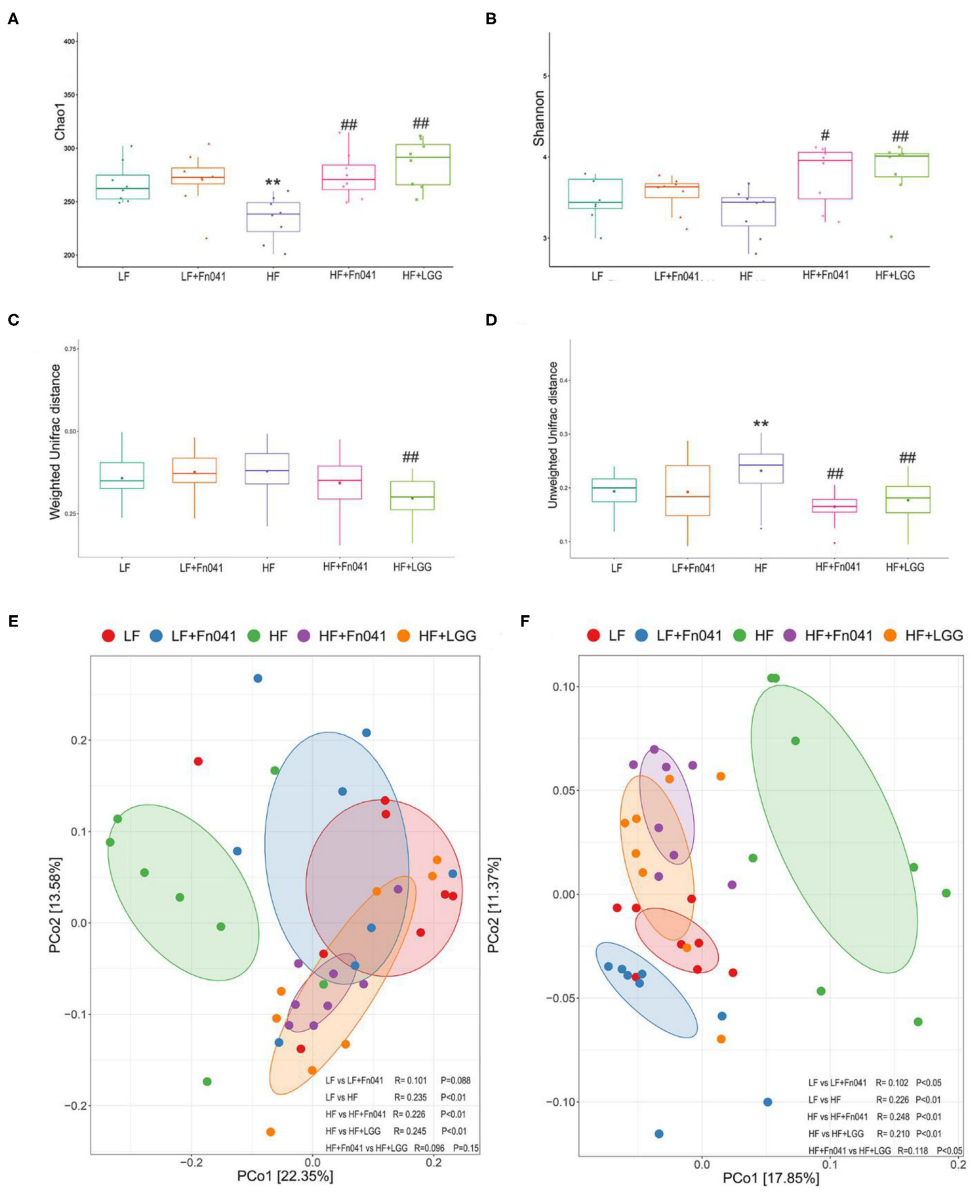
Effect of *Lactobacillus reuteri* Fn041 on species diversity of microbiota in ileum contents. **(A)** Chao index; **(B)** Shannon index; **(C)** Weighted unifrac distance; **(D)** Unweighted unifrac distance; **(E)** Principal coordinate analysis (PCoA) based on weighted unifrac distance. **(F)** PCoA based on unweighted unifrac distance. **p < 0.01 as compared with the LF group; ^#^*p* < 0.05, ^##^*p* < 0.01 as compared with the HF group. (*n* = 8/group). LF, low-fat diet group; LF + Fn041, low-fat diet group treated with *L. reuteri* Fn041; HF, high-fat diet group; HF + Fn041, high-fat diet group treated with *L. reuteri* Fn041; HF + LGG, high-fat diet group treated with *Lactobacillus rhamnosus* GG.

The microbial community richness indicated by the Chao1 estimators showed that the diversity of the HF group was significantly lower than that of the LF group, whereas the diversity levels of the HF+Fn041 and HF+LGG groups were significantly higher than those of the HF group (*p* < 0.01 or *p* < 0.001, Figure 5A). The community diversity estimated by the Shannon index was significantly increased in the HF+Fn041 and HF+LGG groups relative to the HF group (*p* < 0.05, *p* < 0.01, Figure 5B). According to the weighted UniFrac distances of PCo1 (22.35%), there was a significant difference in beta biodiversity between the HF group and HF+LGG (*p* < 0.05, Figure 5C). Combining the unweighted UniFrac distance of PCo1 (17.85%), both Fn041 and LGG induced significant changes in microbiota structure (*p* < 0.001, Figure 5D), which was confirmed by permutational multivariate analysis of variance (both permutational multivariate analysis of variance significances were *p* < 0.01, Figures 5E,F).

### Effect of *L. reuteri* Fn041 on the Composition of the Ileum Microbiota

**Figure 6:**
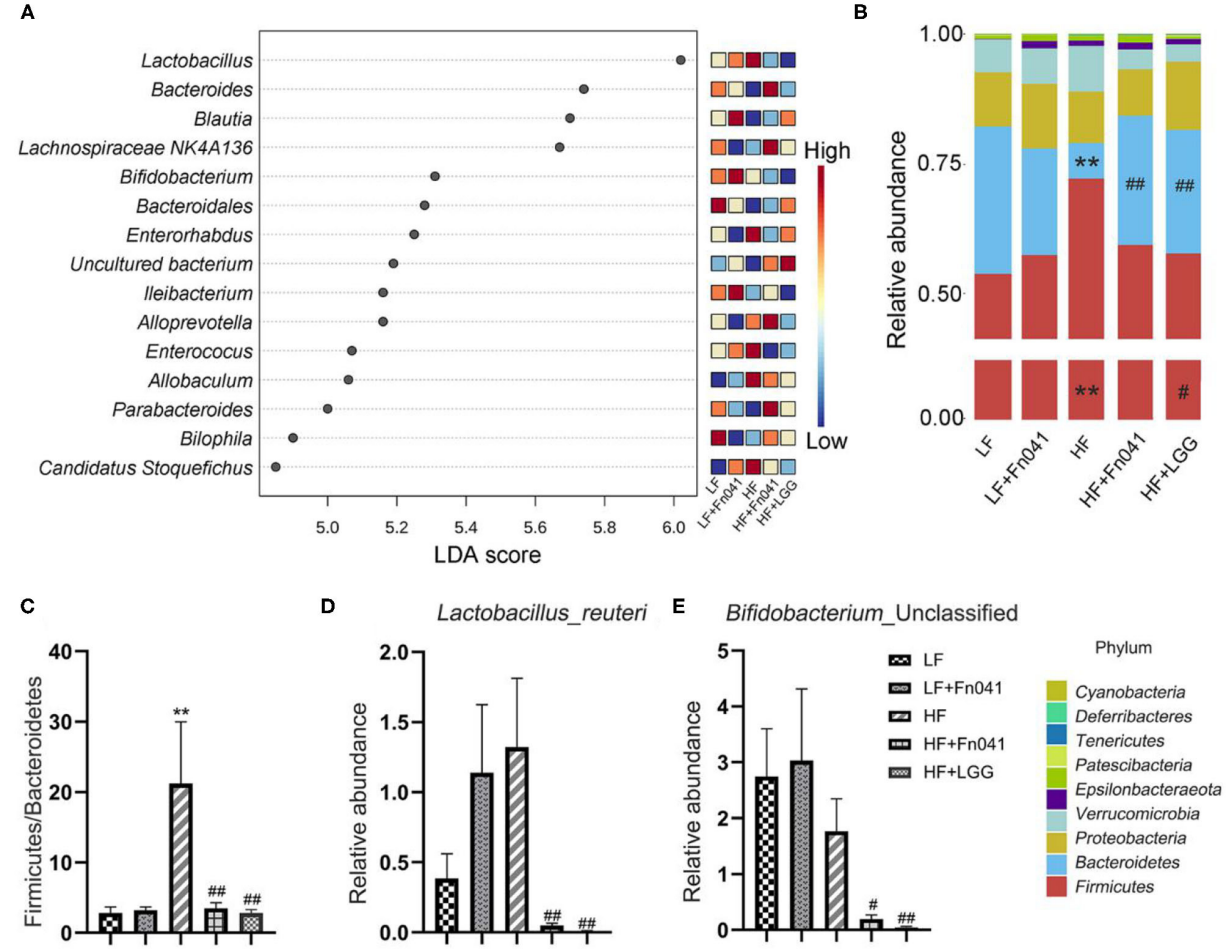
Effect of *Lactobacillus reuteri* Fn041 on structural changes in the gut microbiota. **(A)** LDA combined with effect size measurements (LEfSe) at the genus level. Data with an FDR<0.05 and an LDA score≥4.0 were considered significant in Kruskal–Wallis; **(B)** The bacterial profiles at the phylum level (each color represents one phylum); **(C)** The ratio of *Bacteroidetes* to *Firmicutes*; **(D)** Levels of *L. reuteri*; **(E)** Levels of *Bifidobacteria _*Unclassified. (*n* = 8/group). **p < 0.01 as compared with the LF group; ^#^*p* < 0.05, ^##^*p* < 0.01 as compared with the HF group. LF, low-fat diet group; LF, low-fat diet group; LF + Fn041, low-fat diet group treated with *L. reuteri* Fn041; HF, high-fat diet group; HF + Fn041, high-fat diet group treated with *L. reuteri* Fn041; HF + LGG, high-fat diet group treated with *Lactobacillus rhamnosus* GG.

LDA Effect Size was used to analyze each group of signature genus (Figure 6A). Uncultured *Bacteroidales*_bacterium and *Bilophila* were enriched in the LF group mice, *Bifidobacterium* and *Ileibacterium* were dominant genera in the LF+Fn041 group mice, and *Lactobacillus, Enterorhabdus, Enterococcus, Allobaculum*, and *Candidatus_Stoquefichus* were enriched in the HF group mice. *Bacteroides, Lachnospiraceae*_NK4A136_group, and *Alloprevotella* were the dominant genera in the HF+Fn041 group mice; *Blautia* was enriched in the HF+LGG group (*p* < 0.05, Figure 6A). At the phylum level, the relative abundance of *Bacteroidetes* in the HF group was 21.92% lower than that in the LF group. By comparison, the relative level of *Firmicutes* in the HF group increased by 18.64% compared to that in the LF group (*p* < 0.01, Figure 6B). They recovered to similar levels as the LF group after Fn041 and LGG treatment, respectively (Figure 6B). A significant increase was observed in the ratio of *Firmicutes* to *Bacteroidetes* in the HF group compared to that in the LF group, which was significantly decreased by the Fn041 and LGG treatments (*p* < 0.01, Figure 6C). At the species level, *L. reuteri* and *Bifidobacterium_*Unclassified were significantly lower in the HF+Fn041 and HF+LGG groups than in the HF group (*p* < 0.05, *p* < 0.01, Figures 6D,E).

### Effects of *L. reuteri* Fn041 on Feces Bile Acid and Cholesterol

**Figure 7:**
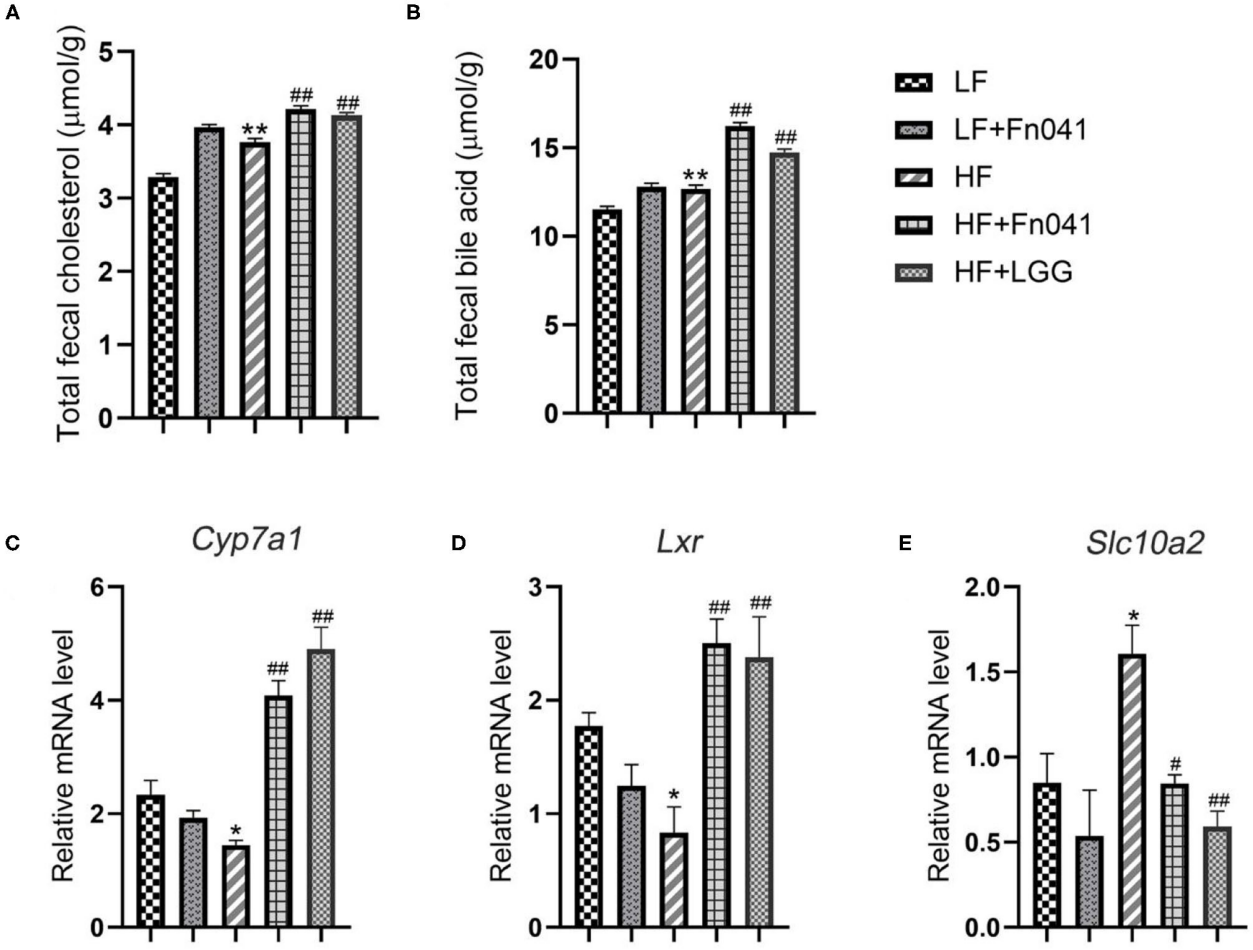
Cholesterol and bile acids in the feces of mice. **(A)** Total cholesterol content in feces; **(B)** total bile acid content in feces; **(C)** liver expression of *Cyp7a1*; **(D)** liver expression of *Lxr*; **(E)** ileum expression of *Slc10a2*; *p < 0.05, **p < 0.01 as compared with the LF group; ^#^*p* < 0.05, ^##^*p* < 0.01 as compared with the HF group. (*n* = 12/group). LF, low-fat diet group; LF + R, low-fat diet group treated with *L. reuteri* Fn041; HF, high-fat diet group; HF + R, high-fat diet group treated with *L. reuteri* Fn041 and *Lactobacillus rhamnosus* GG.

We examined the contents of TC and total bile acids in the feces of mice at 8 weeks and found that the excretion of cholesterol and bile acids in the HF group was significantly higher than that in the LF group. Following the Fn041 and LGG treatments, the excretion rates of cholesterol and bile acids in the HF+Fn041 and HF+LGG groups were significantly higher than those in the HF group (p <0.01, Figures 7A,B). In the HF+Fn041 and HF+LGG groups, the liver *Cyp7a1* and *Lxr* mRNA expression levels were significantly higher than those in the HF group, and the ileal bile transport gene *Slc10a2* mRNA expression level was significantly lower than that in the HF group (*p* < 0.01, Figures 7C–E).

### Association of the Different Genus With Biochemical Indicators and the Relationships of Different Bacteria With Endogenous Metabolites

**Figure 8:**
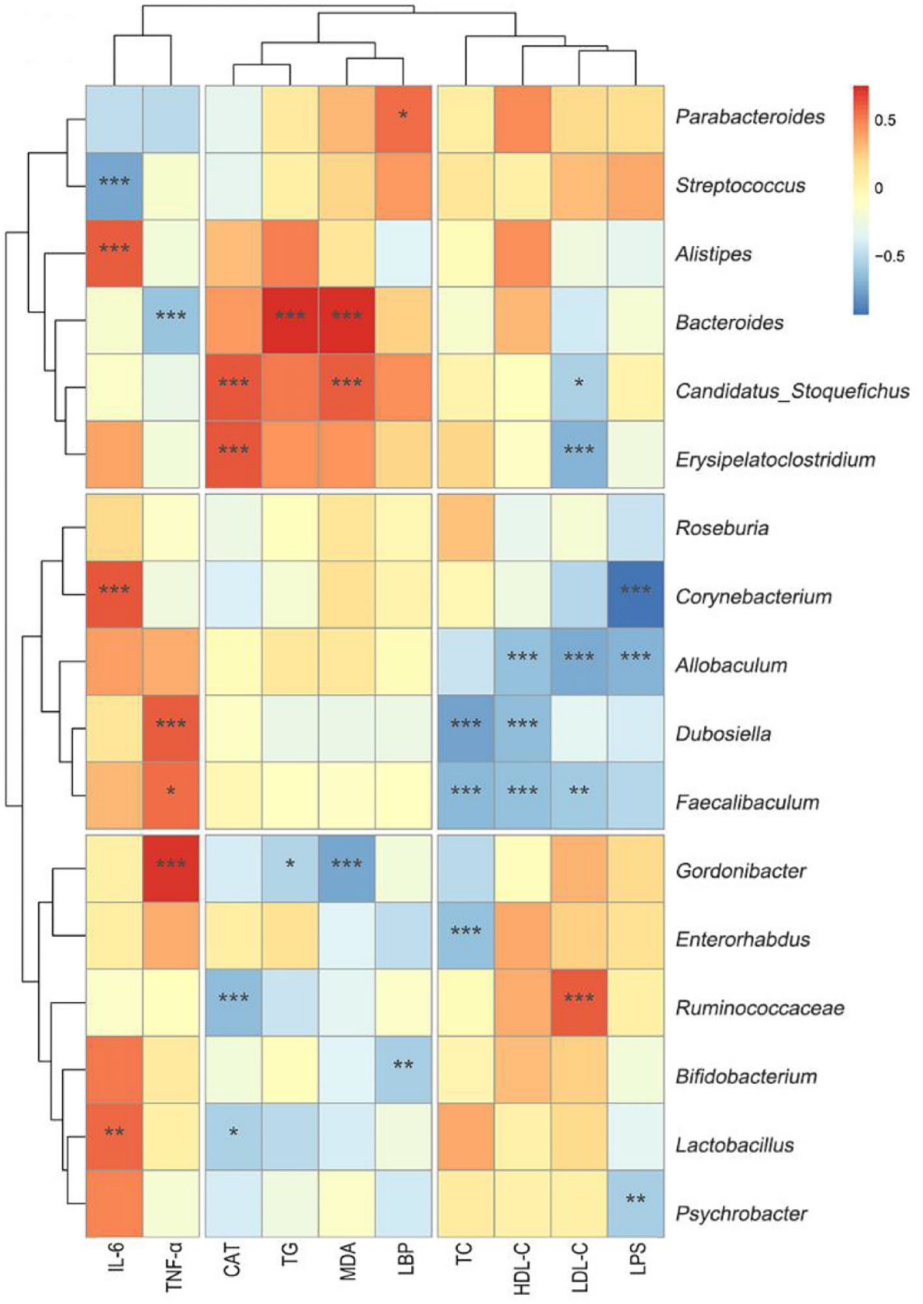
Heat-map of the correlation analysis between different biochemical indicators and genus microbiota. Hierarchical clustering by average linkage of the significantly differentially affected genus as compared HF+Fn041 with the HF group. The differentially affected genus was identified with edgeR; the threshold was set to FDR < 0.05. The color bar indicates the correlation of the indicators (positive correlation, red; negative correlation, blue). *p < 0.05; **p < 0.01; ***p < 0.001 using Spearman correlation. (*n* = 8/group).

As shown in Figure 8, *Erysipelatoclostridium* was negatively correlated with LDL-C (r = −0.68). Corynebacterium was negatively correlated with LPS (r = −0.93). *Allobaculum* was negatively correlated with LPS (r = −0.69), LDL-C (r = −0.72), and HDL-C (r = −0.69). *Dubosiella* was negatively correlated with HDL-C (r = −0.66) and TC (r = −0.75). *Faecalibaculum* was negatively correlated with LDL-C (r = −0.59), HDL-C (r = −0.63), and TC (r = −0.67). *Gordonibacter* (r = −0.78) was negatively correlated with malondialdehyde (r = −0.74). All these correlation coefficients were statistically significant (*p* < 0.01 or *p* < 0.001, Figure 8).

## Discussion

Our study demonstrated that Fn041 prevented high-fat diet-induced hypercholesterolemia, prevented intestinal barrier function damage, and improved intestinal microbiota disorders in mice. The cholesterol-lowering mechanism of action in this study was mainly related to the promotion of bile salt and cholesterol excretion as well as the regulation of intestinal microorganisms and an enhancement of intestinal barrier functions.

In the early 1970's, researchers discovered that a traditional African food fermented by *Lactobacillus* had a serum cholesterol-lowering effect (27). Previous studies have shown that some strains of *L. reuteri* exhibit hypocholesterolemic effects in different animal models. *Lactobacillus reuteri* GMNL-263 ameliorated hyperlipidemia and reduced serum TC and LDL-C levels in hamsters fed a high-fat diet (28). *Lactobacillus reuteri* LR6 reduces serum TC, TG, and LDL-C levels in high-fat diet rats (29). We previously found that Fn041 alleviated hypercholesterolemia in C57BL/6 mice using an experimental intervention model in which 45% of the feed energy was provided by lard (12). In the current study, we have used a prophylactic animal model to demonstrate that Fn041 prevents hypercholesterolemia in C57BL/6 mice, in which 60% of the feed energy is provided by lard. This demonstrates that Fn041 can prevent and treat hypercholesterolemia. Unlike other strains, Fn041 is a breast milk-derived sIgA-coated bacterium that may target binding to the mucus layer in the intestine, thus acting at a closer distance to the intestinal epithelium (30, 31).

In the present study, Fn041 and LGG attenuated a high-fat diet-induced weight gain, demonstrating the efficacy of weight reduction. In general, weight gain is characterized by an increase in intracellular lipid content, adipocyte volume, and the number of adipocytes (32). In this study, Fn041 and LGG reduced high-fat diet-induced lipid accumulations in the adipose tissues of the epididymis and liver, which may at least partially explain their weight loss efficacy. This is consistent with the results of a previous intervention study that demonstrated that Fn041 exerts an inhibitory effect on high-fat diet-induced hepatic steatosis by downregulating *Fas* mRNA expression and upregulating *Srebp1c* mRNA expression (17, 33). LGG has also prevented liver steatosis and adipocyte enlargement caused by a high-fat diet (34). In addition, *L. reuteri* 8513 has attenuated high-fat diet-induced hepatic steatosis in rats (35). This is consistent with the results of the present study.

First, we also attempted to determine the mechanism of action through which Fn041 helps prevent hypercholesterolemia from several aspects. Some studies have shown that germ-free rats tend to accumulate more cholesterol than conventionally housed rats, and that bile acid and cholesterol absorption increase by 300 and 25%, respectively, in the absence of gut microbiota (36). This is consistent with our experimental results. Fn041 and LGG can increase the abundance of intestinal microbiota, indicating that Fn041 can inhibit the absorption of cholesterol. The decrease in cholesterol concentration may be caused by several possible mechanisms, one of which is primarily the degradation of bile salts in the intestine by *Lactobacillus*, followed by the excretion of bile salts in the feces. In this case, the concentration of bile salts decreases, forcing the liver to continue to synthesize bile salts from intrahepatic cholesterol, thereby exerting a cholesterol-lowering effect in the body (18, 19, 37). In contrast, supplementation with *L. reuteri* NCIMB 30242 increased luminal bile salt hydrolase activity, which led to an increase in deconjugate bile acid excretion and a decrease in serum cholesterol levels (38). In another study, *L. reuteri* LR6 deconjugated bile salts, thereby exerting a hypocholesterolemic effect (29). Bile acids in feces are the main product of cholesterol metabolism; therefore, an increase in the excretion of fecal bile acids can reduce serum cholesterol levels (39). Fn041 increased TC and total bile acids in the feces. Activation of the liver CYP7A1I expression may promote the synthesis of bile acids and reduce cholesterol levels in the blood (40). The *Lxr* gene in the liver mainly synthesizes cholesterol in the blood into bile acids by regulating the bile acid synthesis rate enzyme *Cyp7a1*, thereby reducing the cholesterol level in the body (41). This is consistent with our experimental results. The Fn041 and LGG treatment groups upregulated the expression levels of *Lxr* and *Cyp7a1* in the liver and downregulated the expression levels of the ileal bile acid reabsorption transport gene *Slc10a2* (42). This shows that Fn041 and LGG mainly help inhibit bile acid absorption or promote bile acid excretion. In addition, some *L. reuteri* strains produce indole-3-aldehyde (43), indole-3-lactic acid (44), and other unknown aromatic hydrocarbon receptor ligands (45). Serum indole-3-aldehyde is substantially reduced in patients with atherosclerosis, suggesting that it is involved in negative cholesterol regulation (46). Therefore, Fn041 may release indole-3-aldehyde in the intestine and regulate cholesterol homeostasis.

Second, dysbiosis of the gut microbiota caused by a high-fat diet leads to increased intestinal permeability and impairment of intestinal epithelial barrier function (47). A high-fat diet leads to the proliferation of intestinal gram-negative bacteria or Proteobacteria and the release of excess LPS, which may induce intestinal barrier damage by decreasing the expression of tight junction proteins (48). Our previous study confirmed that Fn041 directly regulates the mRNA expression of several tight junction genes, such as Zo1, occludin, and claudin-6, to prevent barrier damage and endotoxemia caused by a high-fat diet (17). LPS is associated with an inflammatory response and is responsible for the pathogenesis of metabolic diseases, as the continuous subcutaneous low rate infusion of LPS induces most features of metabolic diseases (49, 50). For example, LPS injection induces hepatic oxidative stress and disorders of cholesterol metabolism in mice (51). Thus, there is a correlation between endotoxemia, impaired intestinal barrier functions, and host metabolic disorders. This is consistent with the results of our current experiments using plasma FD4 and LPS. We found that Fn041 and LGG alleviated mucosal barrier damage and reduced plasma LPS concentration; therefore, Fn041 prevented high-fat diet-induced barrier damage and endotoxemia (Figure 4).

Third, the gut microbiota and lipid metabolism are mutually regulated. Some studies have shown that a high-fat diet may cause a significant imbalance in the composition of the gut microbiota (52). Compared with a low-fat diet, a high-fat diet reduced the relative abundance of Bacteroides and significantly increased the abundance of *Firmicutes* (53, 54). Our results are in accordance with those of previous studies (Figure 6). We previously showed that Fn041 inhibited a reduction of the Chao 1 index, but had no significant effect on the Shannon index (17). In addition, LGG also inhibited the reduction of the Chao 1 index (34). However, in this study, the Fn041 strain increased the diversity of the gut microbiota and prevented the decrease of Chao 1 and Shannon index, which is in accordance with the results of β-diversity (Figure 5). This shows that Fn041 can alleviate the dysbiosis of gut microbiota caused by a high-fat diet. Studies have shown that a high-fat diet increases bile acid concentrations (55). *L. reuteri* and *Bifidobacteria* demonstrate bile-salt hydrolase activity (34). The fewer the microbes associated with the activity rate of bile-salt hydrolase, the higher the concentration of ileal bound bile acids (56). After the LF group was treated with Fn041, the intestinal *L. reuteri* abundance levels increased significantly, whereas after the HF group was treated, the intestinal abundance levels of *L. reuteri* and *Bifidobacterium* were significantly reduced when the ileal bound bile acid concentration increased. On the one hand, Fn041 acts on the HF group, not by promoting bacterial colonization, but through inhibiting the reabsorption of bile acids in the ileum and promoting the excretion of cholesterol and bile acids (34). On the other hand, an increased intestinal bile concentration inhibits the proliferation of *Lactobacillus* and *Bifidobacterium* (57). Therefore, the levels of bile acids produced by the liver and excreted in the feces increase, and the concentration of cholesterol in the serum decreases. However, increased lumen bile acids may hinder the colonization of *L. reuteri*. *L reuteri* has reportedly been associated with increased body weight (58, 59), and in this study, *L. reuteri* Fn041 reduced intestinal abundance of *L. reuteri*, which may prevent excessive weight gain. Our experimental results showed that Fn041 increased the abundance of *Bacteroides* and *Alloprevotella*. These bacteria can produce short-chain fatty acids, thus lowering cholesterol levels in the body (60, 61). This result is consistent with our previous intervention experiments, where Fn041 increased the number of beneficial bacteria and reduced cholesterol levels in the body by inhibiting hepatic cholesterol synthesis through its metabolites of lactate, propionate, and butyrate (17).

## Conclusions

In summary, our results suggest that Fn041 and LGG can significantly reduce plasma TC and LDL-C levels and prevent liver and testicular fat accumulation. Fn041 and LGG have protective effects on the dysregulation of the intestinal barrier and intestinal microbiota induced by a high-fat diet. In addition, the detection of mouse feces showed that Fn041 promoted the excretion of cholesterol and bile acids. Therefore, cholesterol reduction may be produced by promoting cholesterol excretion and enhancing the mucosal barrier. This study suggests that Fn041 may be a valid candidate for the regulatory function of cholesterol metabolism in foods. A deeper understanding of the molecular pathways by which Fn041 lowers cholesterol levels and changes cholesterol metabolism will potentially open new horizons for the development of related foods to prevent cholesterol metabolism disorders and related intestinal diseases. This would have important industrial implications.

## Data Availability Statement

The datasets presented in this study can be found in online repositories. The names of the repository/repositories and accession number(s) can be found in the article/Supplementary Material.

## Ethics Statement

The animal study was reviewed and approved by the Guidelines for Care and Use of Laboratory Animals of Qingdao University.

## Author Contributions

ML and CQ participated in data collection, performed the analyses, and wrote the paper. JS designed the study, collected the data, and participated in data analysis. YZ, HZ, and XL performed animal experiments. JZho and WH participated in data analysis. JZha, HD, and JS performed microbiota data pre-processing. JS and DL participated in the study design and writing of the paper and was responsible for overall study coordination. All authors contributed to the article and approved the submitted version.

## Funding

This research was supported by the Nutrition Science Research Foundation of BYHEALTH (No. ty202101004) and the Nutrition and Care of Maternal and Child Research Funding program (Grant #2020BINCMCF056).

## Conflict of Interest

The authors declare that the research was conducted in the absence of any commercial or financial relationships that could be construed as a potential conflict of interest.

## Publisher's Note

All claims expressed in this article are solely those of the authors and do not necessarily represent those of their affiliated organizations, or those of the publisher, the editors and the reviewers. Any product that may be evaluated in this article, or claim that may be made by its manufacturer, is not guaranteed or endorsed by the publisher.

## References

[1] Huang H Jiang X Xiao Z Yu L Pham Q Sun J. Red Cabbage Microgreens Lower Circulating Low-Density Lipoprotein (LDL), Liver cholesterol, and Inflammatory Cytokines in Mice Fed a High-Fat Diet. J Agric Food Chem. (2016) 64:9161–71. 10.1021/acs.jafc.6b03805 27933986

[2] Robinson JG Wang S Smith BJ Jacobson TA. Meta-analysis of the relationship between non-high-density lipoprotein cholesterol reduction and coronary heart disease risk. J Am Coll Cardiol. (2009) 53:316–22. 10.1016/j.jacc.2008.10.024 19161879

[3] Gao X Tian Y Randell E Zhou H Sun G. Unfavorable Associations Between Serum Trimethylamine N-Oxide and L-Carnitine Levels with Components of Metabolic Syndrome in the Newfoundland Population. Front Endocrinol (Lausanne). (2019) 10:168. 10.3389/fendo.2019.00168 30972022 PMC6443640

[4] Araújo JR Tomas J Brenner C Sansonetti PJ. Impact of high-fat diet on the intestinal microbiota and small intestinal physiology before and after the onset of obesity. Biochimie. (2017) 141:97–106. 10.1016/j.biochi.2017.05.019 28571979

[5] Ananthakrishnan AN Luo C Yajnik V Khalili H Garber JJ Stevens BW Cleland T Xavier RJ. Gut Microbiome Function Predicts Response to Anti-integrin Biologic Therapy in Inflammatory Bowel Diseases. Cell Host Microbe. (2017) 21:603–610.e3. 10.1016/j.chom.2017.04.010 28494241 PMC5705050

[6] Aminlari L Shekarforoush SS Hosseinzadeh S Nazifi S Sajedianfard J Eskandari MH. Effect of Probiotics Bacillus coagulans and Lactobacillus plantarum on Lipid Profile and Feces Bacteria of Rats Fed Cholesterol-Enriched Diet. Probiotics Antimicrob Proteins. (2019) 11:1163–71. 10.1007/s12602-018-9480-1 30368715

[7] Ron S Shai F Ron M. Are we really vastly outnumbered? revisiting the ratio of bacterial to host cells in humans. Cell. (2016) 164:337–40. 10.1016/j.cell.2016.01.013 26824647

[8] Hasslöf P Stecksén-Blicks C. Chapter 10: Probiotic Bacteria and Dental Caries. Monogr Oral Sci. (2020) 28:99–107. 10.1159/000455377 31940624

[9] Kim K Jeong J Kim D. Lactobacillus brevis OK56 ameliorates high-fat diet-induced obesity in mice by inhibiting NF- κ B activation and gut microbial LPS production. J Funct Foods. (2015) 13:183–91. 10.1016/j.jff.2014.12.045

[10] Bordoni A Amaretti A Leonardi A Boschetti E Danesi F Matteuzzi D. Cholesterol-lowering probiotics: in vitro selection and in vivo testing of bifidobacteria. Appl Microbiol Biotechnol. (2013) 97:8273–81. 10.1007/s00253-013-5088-2 23872958

[11] Taranto MP Medici M Perdigon G Ruiz Holgado AP Valdez GF. Effect of Lactobacillus reuteri on the prevention of hypercholesterolemia in mice. J Dairy Sci. (2000) 83:401–3. 10.3168/jds.S0022-0302(00)74895-8 10750094

[12] Wu Y Zhang Q Ren Y Ruan Z. Effect of probiotic Lactobacillus on lipid profile: A systematic review and meta-analysis of randomized, controlled trials. PLoS ONE. (2017) 12:e0178868. 10.1371/journal.pone.0178868 28594860 PMC5464580

[13] Malpeli A Taranto MP Cravero RC Tavella M Fasano V Vicentin D. Effect of Daily Consumption of Lactobacillus reuteri CRL 1098 on Cholesterol Reduction in Hypercholesterolemic Subjects. Food Nutr Sci. (2015)6:1583–90. 10.4236/fns.2015.617163

[14] Martoni CJ Labbé A Ganopolsky JG Prakash S Jones ML. Changes in bile acids, FGF-19and sterol absorption in response to bile salt hydrolase active L. reuteri NCIMB 30242. Gut Microbes. (2015) 6:57–65. 10.1080/19490976.2015.1005474 25612224 PMC4615650

[15] Ding M Qi C Yang Z Jiang S Bi Y Lai J. Geographical location specific composition of cultured microbiota and Lactobacillus occurrence in human breast milk in China. Food Funct. (2019) 10:554–64. 10.1039/C8FO02182A 30681124

[16] Zhang M Wang LM Chen ZH Zhao ZP Li YC Deng Q. [Multilevel logistic regression analysis on hypercholesterolemia related risk factors among adults in China]. Zhonghua Yu Fang Yi Xue Za Zhi. (2018) 52:151–7. 10.3760/cma.j.issn.0253-9624.2018.02.007 29429269

[17] Li S Qi C Zhu H Yu R Xie C Peng Y. Lactobacillus reuteri improves gut barrier function and affects diurnal variation of the gut microbiota in mice fed a high-fat diet. Food Funct. (2019) 10:4705–15. 10.1039/C9FO00417C 31304501

[18] Jones ML Tomaro-Duchesneau C Martoni CJ Prakash S. Cholesterol lowering with bile salt hydrolase-active probiotic bacteria, mechanism of action, clinical evidence, and future direction for heart health applications. Expert Opin Biol Ther. (2013) 13:631–42. 10.1517/14712598.2013.758706 23350815

[19] Kumar M Nagpal R Kumar R Hemalatha R Verma V Kumar A. Cholesterol-lowering probiotics as potential biotherapeutics for metabolic diseases. Exp Diabetes Res. (2012) 2012:902917. 10.1155/2012/902917 22611376 PMC3352670

[20] Ishimwe N Daliri EB Lee BH Fang F Du G. The perspective on cholesterol-lowering mechanisms of probiotics. Mol Nutr Food Res. (2015) 59:94–105. 10.1002/mnfr.201400548 25403164

[21] Michael DR Moss JWE Calvente DL Garaiova I Plummer SF Ramji DP. Lactobacillus plantarum CUL66 can impact cholesterol homeostasis in Caco-2 enterocytes. Benef Microbes. (2016) 7:443–51. 10.3920/BM2015.0146 26839071

[22] Horáčková Š Plocková M Demnerová K. Importance of microbial defence systems to bile salts and mechanisms of serum cholesterol reduction. Biotechnol Adv. (2018) 36:682–90. 10.1016/j.biotechadv.2017.12.005 29248683

[23] Ji X Shi S Liu B Shan M Tang D Zhang W. Bioactive compounds from herbal medicines to manage dyslipidemia. Biomed Pharmacother. (2019) 118:109338. 10.1016/j.biopha.2019.109338 31545238

[24] Huang Y Zheng Y. The probiotic Lactobacillus acidophilus reduces cholesterol absorption through the down-regulation of Niemann-Pick C1-like 1 in Caco-2 cells. Br J Nutr. (2010) 103:473–8. 10.1017/S0007114509991991 19814836

[25] Gorenjak M Gradišnik L Trapečar M Pistello M Kozmus CP Škorjanc D. Improvement of lipid profile by probiotic/protective cultures: study in a non-carcinogenic small intestinal cell model. New Microbiol. (2014) 37:51–64. 24531171

[26] Huang Y Wu F Wang X Sui Y Yang L Wang J. Characterization of Lactobacillus plantarum Lp27 isolated from Tibetan kefir grains: a potential probiotic bacterium with cholesterol-lowering effects. J Dairy Sci. (2013) 96:2816–25. 10.3168/jds.2012-6371 23498003

[27] Mann G V. Studies of a surfactant and cholesteremia in the Maasai. Am J Clin Nutr. (1974) 27:464–9. 10.1093/ajcn/27.5.464 4596028

[28] Ting WJ Kuo WW Kuo CH Yeh YL Shen CY Chen YH. Supplementary heat-killed Lactobacillus reuteri GMNL-263 ameliorates hyperlipidaemic and cardiac apoptosis in high-fat diet-fed hamsters to maintain cardiovascular function. Br J Nutr. (2015) 114:706–12. 10.1017/S0007114515002469 26234728

[29] Singh TP Malik RK Katkamwar SG Kaur G. Hypocholesterolemic effects of Lactobacillus reuteri LR6 in rats fed on high-cholesterol diet. Int J Food Sci Nutr. (2015) 66:71–5. 10.3109/09637486.2014.953450 25265203

[30] Donaldson GP Ladinsky MS Yu KB Sanders JG Yoo BB Chou WC. Gut microbiota utilize immunoglobulin A for mucosal colonization. Science. (2018) 360:795–800. 10.1126/science.aaq0926 29724905 PMC5973787

[31] Qi C Ding M Li S Zhou Q Li D Yu R. Sex-dependent modulation of immune development in mice by secretory IgA-coated Lactobacillus reuteri isolated from breast milk. J Dairy Sci. (2021) 104:3863–75. 10.3168/jds.2020-19437 33612242

[32] de Ferranti S Mozaffarian D. The perfect storm: obesity, adipocyte dysfunction, and metabolic consequences. Clin Chem. (2008) 54:945–55. 10.1373/clinchem.2007.100156 18436717

[33] Gao X Jiang Y Xu Q Liu F Pang X Wang M. 4-Hydroxyderricin Promotes Apoptosis and Cell Cycle Arrest through Regulating PI3K/AKT/mTOR Pathway in Hepatocellular Cells. Foods (Basel, Switzerland). (2021) 10:2036. 10.3390/foods10092036 34574146 PMC8468691

[34] Joung H Chu J Kim BK Choi IS Kim W Park TS. Probiotics ameliorate chronic low-grade inflammation and fat accumulation with gut microbiota composition change in diet-induced obese mice models. Appl Microbiol Biotechnol. (2021) 105:1203–13. 10.1007/s00253-020-11060-6 33443636

[35] Lew LC Hor YY Jaafar MH Lau ASY Lee BK Chuah LO. Lactobacillus Strains Alleviated Hyperlipidemia and Liver Steatosis in Aging Rats via Activation of AMPK. Int J Mol Sci. (2020) 21:5872. 10.3390/ijms21165872 32824277 PMC7461503

[36] Wostmann BS Wiech NL Kung E. Catabolism and elimination of cholesterol in germfree rats. J Lipid Res. (1966) 7:77–82. 10.1016/S0022-2275(20)39588-2 5900224

[37] Taranto MP Sesma F Valdez GF De Referencia D Cerela L Tucum M De. Localization and primary characterization of bile salt hydrolase from Lactobacillus reuteri. Biotechnol Lett. (1999) 21:935–8. 10.1023/A:1005652501404

[38] Jones ML Martoni CJ Prakash S. Cholesterol lowering and inhibition of sterol absorption by Lactobacillus reuteri NCIMB 30242: a randomized controlled trial. Eur J Clin Nutr. (2012) 66:1234–41. 10.1038/ejcn.2012.126 22990854

[39] Huang WC Chen YM Kan NW Ho CS Wei L Chan CH. Hypolipidemic effects and safety of Lactobacillus reuteri 263 in a hamster model of hyperlipidemia. Nutrients. (2015) 7:3767–82. 10.3390/nu7053767 25988768 PMC4446778

[40] Wahlström A Sayin SI Marschall HU Bäckhed F. Intestinal Crosstalk between Bile Acids and Microbiota and Its Impact on Host Metabolism. Cell Metab. (2016) 24:41–50. 10.1016/j.cmet.2016.05.005 27320064

[41] Chiang JYL. Bile acid regulation of gene expression: roles of nuclear hormone receptors. Endocr Rev. (2002) 23:443–63. 10.1210/er.2000-0035 12202460

[42] Wang L Zhou Y Wang X Zhang G Guo B Hou X. Mechanism of Asbt (Slc10a2)-related bile acid malabsorption in diarrhea after pelvic radiation. Int J Radiat Biol. (2020) 96:510–9. 10.1080/09553002.2020.1707324 31900034

[43] Zelante T Iannitti RG Cunha C De Luca A Giovannini G Pieraccini G. Tryptophan catabolites from microbiota engage aryl hydrocarbon receptor and balance mucosal reactivity via interleukin-22. Immunity. (2013) 39:372–85. 10.1016/j.immuni.2013.08.003 23973224

[44] Cervantes-Barragan L Chai JN Tianero MD Di Luccia B Ahern PP Merriman J. Lactobacillus reuteri induces gut intraepithelial CD4(+)CD8αα(+) T cells. Science. (2017) 357:806–10. 10.1126/science.aah5825 28775213 PMC5687812

[45] Özçam M Tocmo R Oh JH Afrazi A Mezrich JD Roos S. Gut Symbionts Lactobacillus reuteri R2lc and 2010 Encode a Polyketide Synthase Cluster That Activates the Mammalian Aryl Hydrocarbon Receptor. Appl Environ Microbiol. (2019) 85:e01661–18. 10.1128/AEM.01661-18 30389766 PMC6498181

[46] Cason CA Dolan KT Sharma G Tao M Kulkarni R Helenowski IB. Plasma microbiome-modulated indole- and phenyl-derived metabolites associate with advanced atherosclerosis and postoperative outcomes. J Vasc Surg. (2018) 68:1552–62.e7. 10.1016/j.jvs.2017.09.029 29248242 PMC5999545

[47] Fuke N Nagata N Suganuma H Ota T. Regulation of Gut Microbiota and Metabolic Endotoxemia with Dietary Factors. Nutrients. (2019) 11:2277. 10.3390/nu11102277 31547555 PMC6835897

[48] Wu W Wang S Liu Q Shan T Wang Y. Metformin Protects against LPS-Induced Intestinal Barrier Dysfunction by Activating AMPK Pathway. Mol Pharm. (2018) 15:3272–84. 10.1021/acs.molpharmaceut.8b00332 29969038

[49] Cheng XR Guan LJ Muskat MN Cao CC Guan B. Effects of Ejiao peptide-iron chelates on intestinal inflammation and gut microbiota in iron deficiency anemic mice. Food Funct. (2021) 12:10887–902. 10.1039/D1FO01802G 34643632

[50] Cani PD Bibiloni R Knauf C Waget A Neyrinck AM Delzenne NM. Changes in gut microbiota control metabolic endotoxemia-induced inflammation in high-fat diet-induced obesity and diabetes in mice. Diabetes. (2008) 57:1470–81. 10.2337/db07-1403 18305141

[51] El Kamouni S El Kebbaj R Andreoletti P El Ktaibi A Rharrassi I Essamadi A. Protective Effect of Argan and Olive Oils against LPS-Induced Oxidative Stress and Inflammation in Mice Livers. Int J Mol Sci. (2017) 18:2181. 10.3390/ijms18102181 29048364 PMC5666862

[52] Murphy EA Velazquez KT Herbert KM. Influence of high-fat diet on gut microbiota: a driving force for chronic disease risk. Curr Opin Clin Nutr Metab Care. (2015) 18:515–20. 10.1097/MCO.0000000000000209 26154278 PMC4578152

[53] Hu H Zhang S Liu F Zhang P Muhammad Z Pan S. Role of the Gut Microbiota and Their Metabolites in Modulating the Cholesterol-Lowering Effects of Citrus Pectin Oligosaccharides in C57BL/6 Mice. J Agric Food Chem. (2019) 67:11922–30. 10.1021/acs.jafc.9b03731 31576748

[54] Chen K Chen H Faas MM de Haan BJ Li J Xiao P. Specific inulin-type fructan fibers protect against autoimmune diabetes by modulating gut immunity, barrier function, and microbiota homeostasis. Mol Nutr Food Res. (2017) 61:1248–9. 10.1002/mnfr.201601006 28218451

[55] Wan Y Yuan J Li J Li H Zhang J Tang J. Unconjugated and secondary bile acid profiles in response to higher-fat, lower-carbohydrate diet and associated with related gut microbiota: a 6-month randomized controlled-feeding trial. Clin Nutr. (2020) 39:395–404. 10.1016/j.clnu.2019.02.037 30876827

[56] Huang F Zheng X Ma X Jiang R Zhou W Zhou S. Theabrownin from Pu-erh tea attenuates hypercholesterolemia via modulation of gut microbiota and bile acid metabolism. Nat Commun. (2019) 10:4971. 10.1038/s41467-019-12896-x 31672964 PMC6823360

[57] Hou G Peng W Wei L Li R Yuan Y Huang X. Lactobacillus delbrueckii Interfere With Bile Acid Enterohepatic Circulation to Regulate Cholesterol Metabolism of Growing-Finishing Pigs via Its Bile Salt Hydrolase Activity. Front Nutr. (2020) 7:617676. 10.3389/fnut.2020.617676 33363199 PMC7759492

[58] Million M Maraninchi M Henry M Armougom F Richet H Carrieri P. Obesity-associated gut microbiota is enriched in Lactobacillus reuteri and depleted in Bifidobacterium animalis and Methanobrevibacter smithii. Int J Obes. (2012) 36:817–25. 10.1038/ijo.2011.153 21829158 PMC3374072

[59] Chan YK Brar MS Kirjavainen PV Chen Y Peng J Li D. High fat diet induced atherosclerosis is accompanied with low colonic bacterial diversity and altered abundances that correlates with plaque size, plasma A-FABP and cholesterol: a pilot study of high fat diet and its intervention with Lactobacillus rhamn. BMC Microbiol. (2016) 16:264. 10.1186/s12866-016-0883-4 27821063 PMC5100306

[60] Zhou L Xiao X Zhang Q Zheng J Deng M. Maternal Genistein Intake Mitigates the Deleterious Effects of High-Fat Diet on Glucose and Lipid Metabolism and modulates Gut Microbiota in Adult Life of Male Mice. Front Physiol. (2019) 10:985. 10.3389/fphys.2019.00985 31417434 PMC6682633

[61] Li TT Liu YY Wan XZ Huang ZR Liu B Zhao C. Regulatory Efficacy of the Polyunsaturated Fatty Acids from Microalgae Spirulina platensis on Lipid Metabolism and Gut Microbiota in High-Fat Diet Rats. Int J Mol Sci. (2018) 19:3075. 10.3390/ijms19103075 30304774 PMC6213792

