# Supplementary material for: Prevention of High-Fat Diet-Induced Hypercholesterolemia by Lactobacillus reuteri Fn041 Through Promoting Cholesterol and Bile Salt Excretion and Intestinal Mucosal Barrier Functions
Source: Front Nutr. 2022 Mar 11;9:851541. doi: 10.3389/fnut.2022.851541 (PMC8967143; doi:10.3389/fnut.2022.851541)
Supplement: Supplementary file 1 [file Table_1.DOCX]

Supplementary Material

# Supplementary Tables

Table S1 Feed composition of mice

| composition | Low fat feed | | High fat feed | |
| --- | --- | --- | --- | --- |
|  | g% | kcal% | g% | kcal% |
| Casein, 30 mesh | 18.96 | 19.72 | 25.85 | 19.72 |
| L-cystine | 0.28 | 0.3 | 0.39 | 0.3 |
| Corn starch | 29.86 | 31.06 | 0 | 0 |
| Maltodextrin | 3.32 | 3.45 | 16.15 | 12.32 |
| Sucrose | 33.18 | 34.51 | 8.89 | 6.78 |
| Microcrystalline cellulose | 4.74 | 0 | 6.46 | 0 |
| Soybean oil | 2.37 | 5.55 | 3.23 | 5.55 |
| Lard | 1.9 | 4.44 | 31.66 | 54.35 |
| Mineral mixture | 0.95 | 0 | 1.29 | 0 |
| Dicalcium phosphate | 1.23 | 0 | 1.68 | 0 |
| Calcium carbonate | 0.52 | 0 | 0.71 | 0 |
| Potassium citrate monohydrate | 1.56 | 0 | 2.13 | 0 |
| Vitamins mixture | 0.95 | 1 | 1.29 | 0.99 |
| Choline hydrogen tartrate | 0.19 | 0 | 0.26 | 0 |
| Energy (kcal/g) |  | 3.85 |  | 5.24 |
